# Web Camera Based Eye Tracking to Assess Visual Memory on a Visual Paired Comparison Task

**DOI:** 10.3389/fnins.2017.00370

**Published:** 2017-06-28

**Authors:** Nicholas T. Bott, Alex Lange, Dorene Rentz, Elizabeth Buffalo, Paul Clopton, Stuart Zola

**Affiliations:** ^1^Department of Medicine, School of Medicine, Stanford UniversityStanford, CA, United States; ^2^Neurotrack Technologies, Inc.Redwood City, CA, United States; ^3^Department of Neurology, Massachusetts General HospitalBoston, MA, United States; ^4^Department of Neurology, Brigham and Women's HospitalBoston, MA, United States; ^5^Department of Physiology and Biophysics, University of WashingtonSeattle, WA, United States; ^6^San Diego Veteran's Administration Medical Center, San DiegoSan Diego, CA, United States; ^7^Emory University Office of the ProvostAtlanta, GA, United States

**Keywords:** web-camera, eye tracking, visual paired comparison, visual memory, methodology comparison

## Abstract

**Background:** Web cameras are increasingly part of the standard hardware of most smart devices. Eye movements can often provide a noninvasive “window on the brain,” and the recording of eye movements using web cameras is a burgeoning area of research.

**Objective:** This study investigated a novel methodology for administering a visual paired comparison (VPC) decisional task using a web camera.To further assess this method, we examined the correlation between a standard eye-tracking camera automated scoring procedure [obtaining images at 60 frames per second (FPS)] and a manually scored procedure using a built-in laptop web camera (obtaining images at 3 FPS).

**Methods:** This was an observational study of 54 clinically normal older adults.Subjects completed three in-clinic visits with simultaneous recording of eye movements on a VPC decision task by a standard eye tracker camera and a built-in laptop-based web camera. Inter-rater reliability was analyzed using Siegel and Castellan's kappa formula. Pearson correlations were used to investigate the correlation between VPC performance using a standard eye tracker camera and a built-in web camera.

**Results:** Strong associations were observed on VPC mean novelty preference score between the 60 FPS eye tracker and 3 FPS built-in web camera at each of the three visits (*r* = 0.88–0.92). Inter-rater agreement of web camera scoring at each time point was high (κ = 0.81–0.88). There were strong relationships on VPC mean novelty preference score between 10, 5, and 3 FPS training sets (*r* = 0.88–0.94). Significantly fewer data quality issues were encountered using the built-in web camera.

**Conclusions:** Human scoring of a VPC decisional task using a built-in laptop web camera correlated strongly with automated scoring of the same task using a standard high frame rate eye tracker camera.While this method is not suitable for eye tracking paradigms requiring the collection and analysis of fine-grained metrics, such as fixation points, built-in web cameras are a standard feature of most smart devices (e.g., laptops, tablets, smart phones) and can be effectively employed to track eye movements on decisional tasks with high accuracy and minimal cost.

## Introduction

Web cameras are increasingly part of the standard hardware of most smart devices. The quality and cost of these devices has allowed for their increased use worldwide and are now a standard feature on most smart devices, including desktop and laptop computers, tablets, and smart phones. Because eye movements can often provide a noninvasive “window on the brain,” the recording of eye movements using web cameras is a burgeoning area of research including both online and offline system development (Wang and Sung, 2001; Hansen and Pece, 2005; Vivero et al., 2010; Anderson et al., 2011; Lin et al., 2013; Petridis et al., 2013).

Visual paired comparison (VPC) task paradigms assess recognition memory through comparison of the proportion of time an individual spends viewing a new picture compared to a picture they have previously seen, i.e., a novelty preference (Fantz, 1964; Fagan, 1970). A novelty preference, or more time spent looking at the new picture, is expected in individuals with normal memory function. By contrast, individuals with memory difficulties are characterized by more equally distributed viewing times between the novel and familiar pictures. The lack of novelty preference suggests impaired declarative memory for what has already been viewed. VPC tasks have been shown to reliably detect memory dysfunction in both primates and humans—both infant and adult (Gunderson and Sackett, 1984; Gunderson and Swartz, 1985; Bachevalier et al., 1993; Pascalis et al., 1998; Manns et al., 2000; Zola et al., 2000, 2013; Crutcher et al., 2009).

Traditionally, VPC task data is captured using a commercial grade eye tracker, employing a high frame rate camera capable of capturing a number of visual features. These data are analyzed using software provided by the manufacturer or are inspected manually by researchers with expertise in the evaluation of eye tracking metrics (e.g., gaze fixation, saccades, blinks, etc.) The cost and complexities involved with commercial grade eye tracking devices have prevented eye tracking from being incorporated into routine clinical assessment; instead, they have remained largely a feature of clinical research. Advances in hardware and software have made web camera eye tracking more affordable and accessible. In addition to the vastly lower price compared to research eye-tracking cameras, which can cost more than $50,000, built-in web cameras do not require the same amount of setup and maintenance, which are necessary for satisfactory data collection from research eye tracking cameras. Furthermore, the accuracy and integrity of data acquired through research eye tracking cameras varies widely (17). Perhaps the most significant advantage of built-in web cameras is the lack of geographical restriction to collect eye feature data on large samples sizes. For example, open source eye tracking software, such as WebGazer.js [https://webgazer.cs.brown.edu/] can be deployed across most major web browsers. At the same time, validation of specific web camera based eye tracking paradigms for assessment of specific cognitive functions remains lacking.

The purpose of this study was to compare the accuracy of human-coded gaze positions on a VPC task in healthy older adults using a laptop-based web camera to a commercially available high-frame-rate eye-tracking camera. By using a built-in web camera it is possible to extend the accessibility of specific eye tracking tasks to anyone with such a device. Similarly, the use of a web camera increases the convenience of data acquisition. In this study we report this method's utility for tracking human eye movements on a VPC decisional task and demonstrate the equivalence of accuracy between this method and that of a commercial high frame rate eye-tracking camera.

## Materials and methods

After detailing the system components for both the standard eye tracking camera system and the web-camera, we will detail the test construction of the VPC task, including the visual calibration, data acquisition, and scoring methods associated with each eye tracking system. Descriptions of the subject sample, procedures and data analysis will conclude this section.

### System components

Subject's eye movements were recorded using a Tobii X2-60 eye tracker camera system (Tobii AB, Stockholm) and using a built-in web camera on a 13-inch Apple Macbook Air laptop (Apple, Cupertino, CA).

#### Eye tracker camera

The system sampled at 60 Hz and the gaze angle was determined by the relative positions of corneal and pupil centers. Participants were seated ~ 27 inches from a 19-inch flat panel monitor that displayed the stimuli. Eye data were recorded using the Tobii SDK and API.

#### Web camera

The laptop processor was a 1.4 GHz Intel Core i5 with 4 GB 1,600 MHz DDR3 memory and a 1,536 MB Intel HD Graphics 5,000 Graphics card. Video resolution of the laptop during test recording was 640 × 480.

### Test construction

Visual paired-comparison tasks make use of familiar and novel visual stimuli. A typical VPC procedure involves a familiarization phase and a test phase. During the initial familiarization phase, subjects were presented with pairs of identical visual stimuli for a fixed period of time. During the test phase, which follows a delay of either fixed or variable periods of time, subjects were presented with additional pairs of visual stimuli, which includes one stimulus from the familiarization phase (familiar stimulus) and a new, or novel, stimulus. The ratio of time subjects spend looking at the novel stimulus relative to total viewing time during the test produced a novelty preference score.

**Figure 1:**
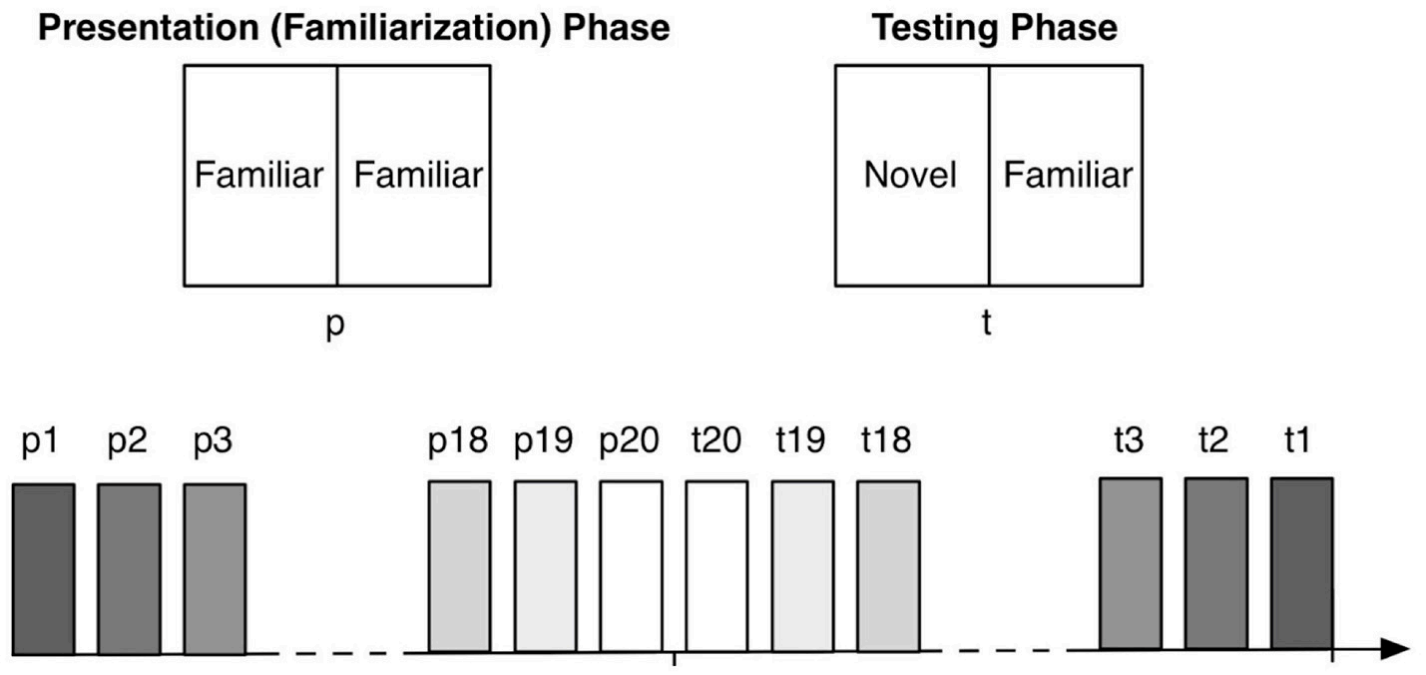
Schematic of familiarization and testing phase stimuli presentation (top) and VPC decisional task familiarization phase (p) and testing phase (t) trial order.

The construction of the VPC task used in this study was a 5-min adapted version of a 30-min VPC task developed by Zola et al. (2013). The test was made up of 20 trials, each trial containing a familiarization phase and a test phase. Similar to the Zola and colleagues task paradigm, the familiarization and test phases were not consecutive (see Figure 1).

**Figure 2:**
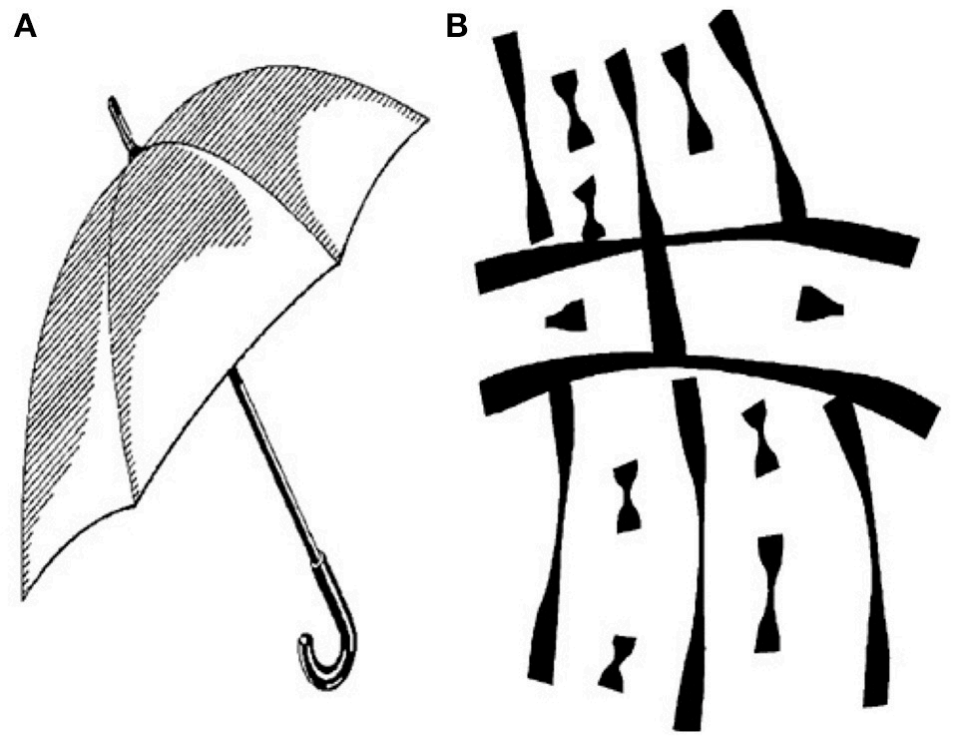
Example VPC stimuli **(A)** nameable, **(B)** unnamable.

During the familiarization phase, the participant was shown a sequence of pairs of identical images. Later on in the test, the test phase occurs, where the participant was shown another sequence of pairs of images, each pair consisting of an image from the familiarization phase, and a novel image. The test phases were shown after all familiarization phases, and in reverse order. Stimuli consisted of black and white, high contrast images measuring 4.4 inches wide by 6.5 inches high. Unique images were used for each trial (Figure 2).

### Calibration validation

Before the start of the exam subjects were instructed to watch a blue dot move around the screen. With the computer screen interpreted as a coordinate system, where the top left of the screen is (0, 0) and the bottom right of the screen is (1, 1), the movement of the ball for calibration was:

(0.5, 0.5)-> (0.1, 0.1)-> (0.1, 0.9)-> (0.9, 0.9)-> (0.5, 0.5)-> (0.9, 0.1)-> (0.5, 0.1)-> (0.1, 0.5)-> (0.5, 0.9),

At each of the above points the ball paused and pulsed for ~2 s.

#### Eye tracking camera

At the conclusion of the calibration phase, two values were computed to use as accuracy measures. They were:

1. Root-mean-square error (RMSE), where the error for each gaze point is computed as Euclidean distance between the point and the position of the calibration ball.
2. Quality, a value computed from the validity codes given by the Tobii X2-60 SDK/API.

If either value was below a certain threshold (0.10 for the first calibration and 0.15 for the second calibration for RMSE and 0.8 for the first calibration and 0.7 for the second calibration for quality), the calibration was repeated up to two additional times in order to ensure accurate evaluation of gaze position. Calibration data were not incorporated into the experimental procedure.

#### Web camera

**Figure 3:**
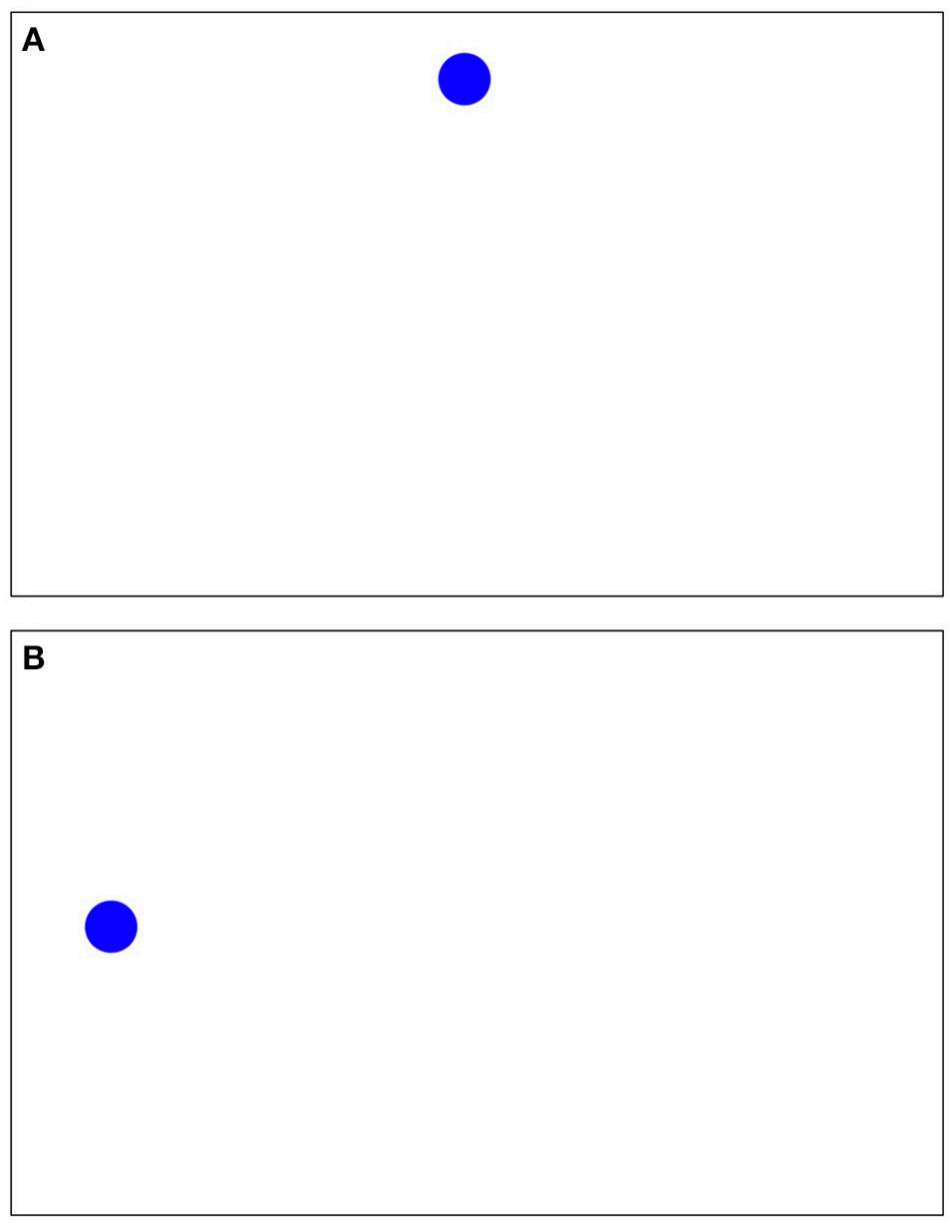
Calibration phase stimuli and location **(A)** top center, **(B)** moving to middle left.

Three human coders manually evaluated the individual frames of the calibration-phase video. All calibration frames (30/s) were evaluated. Validation of the individual calibration frames was accomplished by counting and comparing the frequency of frames coded as the same side as the corresponding position of the calibration dot. Only “left” or “right” were compared to when the ball was either left or right. If individual accuracy of correctly coded calibration frames did not reach 90% or greater, the coding of the calibration-phase video was repeated. Frames when the ball was pulsing in the center (vertically top, middle, and bottom) of the screen were excluded (Figure 3). Calibration data were not incorporated into the experimental procedure.

### Data acquisition

#### Eye tracking camera

Subjects were recorded during the calibration and test phase with a Tobii X2-60 eye tracking device which recorded eye gaze data at 60 Hz (60/s). We programmatically accessed the raw Tobii data using the Tobii Pro Analytics SDK. Each data point recorded by the Tobii contained eye gaze data in two separate “coordinate systems,” the Active Display Coordinate System (ADCS) and the User Coordinate System (UCS). The ADCS is a 2D coordinate system that was configured so that it is aligned with the laptop screen. The top-left corner of the screen is the ADCS coordinate (0, 0); the bottom-right corner is (1, 1). The UCS is a 3D coordinate system that provided additional data on the real-world position of the participant's eyes, allowing us to, for example, accurately estimate the distance from the eye to the screen and thus the degree of visual angle (DVA) for each participant. Each data point consisted of an estimated gaze point for both the left and right eye. For each such datum, we took the midpoint of the two gaze points as the definitive gaze estimate. This was also the default setting in Tobii Pro Studio.

In addition to coordinate data, each Tobii data point contained a “validity code,” which was used to estimate “how certain the eye tracker is that the data given for an eye really originates from that eye.” The code can range from 0 (eye definitely found) to 4 (eye not found). If both eyes were given a validity code of 4, the data point was thrown out. Test trials were automatically excluded if more than 4 s of data was excluded due to poor validity codes.

##### Fixation filter.

We developed a fixation filter in order to process the raw Tobii data. This filter replicated the cluster-based algorithm used by Zola et al. (2013). The original algorithm consisted of the following two phases:

1. Find a sequence of points of a minimum duration 100 ms such that the standard deviation in both the horizontal and vertical directions is at most 0.5 ^*^ 1 DVA. This is the start of a fixation.
2. Continue adding consecutive points to this fixation cluster until 3 such points are at least 1 DVA away from the initial fixation in either the horizontal or vertical direction.

Implementation and processing of the raw gaze data from the Zola study reproduced the same fixation/saccade categorization that was computed and used previously. In phase 2, a fixation ended after 3 points were distant from the fixation in an attempt to filter out the noise in the gaze data by ignoring anomalies. For example, if a single gaze point was estimated incorrectly such that it was significantly distant from its neighboring gaze points it would not necessarily end the fixation.

The Zola study used an *Applied Science Laboratories* eye tracker that recorded gaze data at 120 Hz (120/s). As the Tobii X2-60 records at half this rate, it was necessary to adjust the constants used in the initial algorithm. We experimented with a number of different constants, and examined the individual scan paths of the calibration and tests to determine which remained accurate. The final algorithm consisted of the following rules:

1. Start of fixation: Find a sequence with a minimum of 4 points (~66 ms duration) such that the standard deviation in both the horizontal and vertical directions is at most 1 DVA.
2. End of fixation: Continue adding consecutive points to this fixation cluster until 3 such points are at least 1 DVA away from the initial fixation.

The default setting for the minimum fixation duration in the Tobii I-VT Fixation Filter is 60 ms, which was equivalent to the algorithm we developed (~66 ms). Three researchers with expertise in eye tracking behavior and the Tobii X2-60 eye tracker system independently inspected all test trials to ensure the quality of test data. Test trials flagged for aberrant gaze paths (e.g., gaze clustering, erratic saccades, etc.) were discussed corporately and a consensus decision was made to retain or discard the trial in question. If 10 or more trials were discarded, data for that subject was left out of analyses.

#### Web camera

Subjects were recorded during the calibration and test via the laptop webcam. We used a high definition Flash video recorder (HDFVR) for the subject-side recording, and the resulting Flash video (FLV) video was streamed to our own Wowza Amazon Web Services (AWS) instance. During both calibration and testing, metadata was injected into the FLV video to ensure correspondence between frames of the video and events of the test. In addition, individual trial data was stored in a secure database. Examples of metadata include the times when the calibration ball moves from one coordinate to the next, and when images of the exam are shown and hidden.

### Scoring

The primary performance metric for VPC tasks is novelty preference, which in the present study was the percentage of time the participant spends looking at the novel image compared with the familiar image. Thus, the novelty preference score for each test trial was calculated as (Time viewing novel image)/(Total time viewing either image) The final novelty preference score of a full test was the mean novelty preference score of all 20 trials.

#### Eye tracking camera

For each trial, we established a rectangular area-of-interest perimeter around each of the paired images. This rectangle was of fixed size, and slightly larger than the image it encompasses in order to allow for some error in the gaze data. The novelty preference of each trial was computed as:

(Time fixated on novel image)/(Total time fixated on either image),

Time spent viewing images was calculated based upon the total gaze fixation time recorded by the Tobii X2-60 software.

#### Web camera human-coded scoring

**Figure 4:**
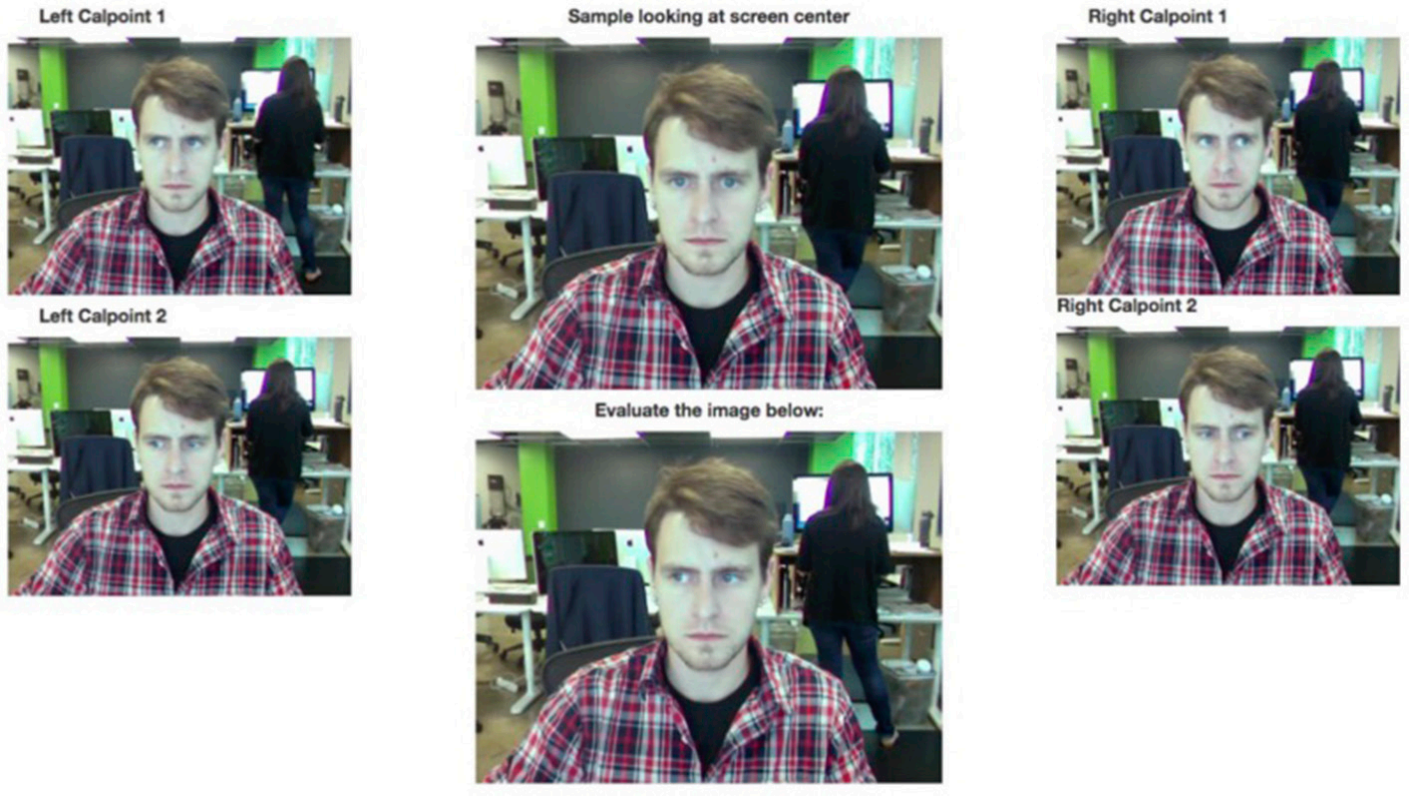
Scoring interface with 2 examples of subject right eye gaze (left calibration points 1 and 2 from the scorer's perspective), 2 examples of subject left eye gaze (right calibration points 1 and 2 from the scorer's perspective), example of subject looking at screen center (top center), and individual subject trial image to be scored (bottom center).

Recorded web camera data was separated into individual frames. The frames were sorted and categorized by test trial using the injected metadata of the FLV file during video acquisition. Web camera data was analyzed using three different frame rates: 10 frames per second (FPS), 5 FPS, and 3 FPS. Processed data was then evaluated on a frame by frame basis by 3 independent human coders to determine if the subject was looking to the left or right side of the screen, or in neither direction. Coding of the “neither” option was intended for frames when the participant was blinking, or the image was of poor enough quality that the iris was indistinguishable from the rest of the eye (Figure 4).

In other words, the human coders were instructed to pick left or right even when it appeared as though the participant was looking more toward the center of the screen (because they were likely not actually looking at the center).

For each image, the majority decision was taken by the individual ratings. For example, if one coder rated the image as “left” while the other two rated the image as “right,” the final rating would be “right.” Then, for each trial, the merged “left” and “right” ratings were translated to “novel” and “familiar” ratings based on which image of the pair is novel. The *novelty preference score* for each trial was the percentage of frames that the participant was rated as looking at the novel side.

Novelty Preference = (# of “novel” frames)/(total # of “novel” frames + # of “familiar” frames).

#### Subjects

Fifty-four clinically normal, community-dwelling, older adults were recruited from volunteers interested in research studies at the Center for Alzheimer Research and Treatment at Brigham and Women's Hospital and at the Massachusetts Alzheimer Disease Research Center at Massachusetts General Hospital. The study was approved by the Partners Human Research Committee, the Institutional Review Board (IRB) for Brigham and Women's Hospital and Massachusetts General Hospital. Subjects underwent informed consent procedures approved by the IRB and all subjects provided written and informed consent for participation in the study. Exclusion criteria included a history of alcoholism, drug abuse, head trauma or current serious medical or psychiatric illnesses. All subjects met inclusion criteria of age (above 50 years old), and cognitive status via score within age-specified norms on the Telephone Interview of Cognitive Status (TICS) (Knopman et al., 2010). No prior computer knowledge was required.

### Subject characteristics

Subjects were all cognitively normal community-dwelling individuals. Mean age was 68.7 ± 7.6 (range: 54–97). The sample was 58.5% female (31 subjects). Mean years of education completed were 15.6 ± 2.8 (range: 12–20). The sample was 57.4% European-American (31 subjects) and 42.6% African-American (23 subjects). See Table 1. Due to subject attrition and technical issues, data from 44 subjects was available at time point 1, data from 36 subjects was available at time point 2, and data from 38 subjects was available at time point 3.

**Table 1:** Subject characteristics.

	**% *M* (SD)**
**DEMOGRAPHICS**	
Sex (% Female)	58.5
Age (years)	68.7 (7.6)
Education (years)	15.6 (2.8)
Race (% Black)	54.7

#### Procedures

Subjects were asked to take part in three in-clinic visits as part of a larger research protocol investigating longitudinal performance on paper-pencil and digitally based cognitive tests. The second visit occurred 1 week after the first visit, and the third visit occurred 6 weeks after the first visit. At the first visit, subjects were administered the original 30-min VPC task using the standard eye tracking camera and the adapted 5-min version with simultaneous capture using both the standard eye tracking camera and the built-in web camera. At subsequent visits, only the 5-min version was administered. At the start of each VPC task administration the subjects were told that images would appear on the screen and that they should look at the images “as if watching television.”

### Data analysis

Analyses were conducted using IBM SPSS version 21.0. Inter-rater agreement of web camera scoring at each time point was assessed using Siegel and Castellan's kappa calculation (Hallgren, 2012). Reliability within the web camera was assessed using Pearson's product-moment correlations. Training sets using 10, 5, and 3 FPS data were analyzed using a sub sample of the participants (*n* = 25). Reliability between the Tobii X2-60 eye tracking camera and the web camera using 3 FPS was assessed using Pearson's product-moment correlations. Cohen's standard was used to determine the strength of these relationships with correlation coefficients of around 0.10 as small, 0.30 as medium, and 0.50 and above as large (Cohen, 1992). Chi-square analyses assessed the frequency of data quality issues present across each of the three time points, and the frequency of data quality issues associated with the eye tracker camera and the built-in web camera.

## Results

### Human coder scoring agreement of web camera data

Analysis of inter-rater agreement of web camera scoring at each time point using Siegel and Castellan's kappa formula revealed very good mean agreement across each of the three human raters for each of the 20 test trials at time points 1–3 (κ = 0.85, 0.88, 0.81, respectively).

### Relationships between web camera FPS training sets

Analysis of relationship between the 10, 5, and 3 FPS training sets revealed robust, positive associations between each FPS training set. Pearson's product-moment correlation between 5 and 10 FPS training sets was 0.91 (*n* = 25; *p* < 0.001). Pearson's product-moment correlation between 3 and 10 FPS training sets was 0.94 (*n* = 25; *p* < 0.001). Pearson's product-moment correlation between 3 and 5 FPS training sets was 0.88 (*n* = 25; *p* < 0.001).

### Relationships between eye tracker camera and web camera

**Figure 5:**
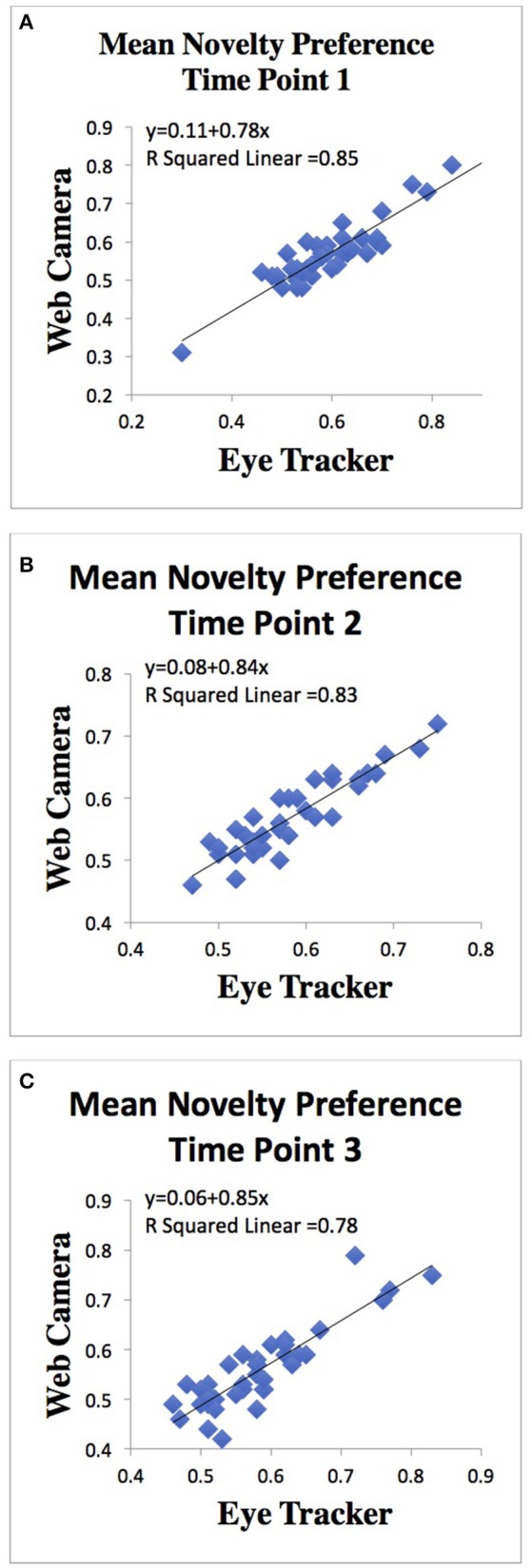
Scatterplots of the relationships between **(A)** Mean novelty preference of 60 FPS eye tracker camera and mean novelty preference of 3 FPS built-in web camera at time 1, **(B)** Mean novelty preference of 60 FPS eye tracker camera and mean novelty preference of 3 FPS built-in web camera at time 2, and **(C)** Mean novelty preference of 60 FPS eye tracker camera and mean novelty preference of 3 FPS built-in web camera at time point 3.

Given the strength of the correlation between the 3 FPS web camera training set and both the 5 and 10 FPS training sets, the 3 FPS data was used to investigate the relationship between the eye-tracker and web camera data. Analysis of the relationship between the eye-tracker and built-in web camera data revealed robust positive associations between each camera type at each time point. Pearson's product-moment correlation at time point 1 was 0.92 (*n* = 44; *p* < 0.001). Pearson's product-moment correlation at time point 2 was 0.91 (*n* = 36; *p* < 0.001). Pearson's product-moment correlation at time point 3 was 0.88 (*n* = 38; *p* < 0.001; Figure 5).

#### Trial level relationships between eye tracker camera and web camera

To further investigate the relationship between the eye tracker camera and the web camera each of the 20 test trials for each time point were compared. Analyses revealed good associations between each camera type at each time point. Pearson's product-moment correlation at time point 1 was 0.76 (*n* = 841; *p* < 0.001). Pearson's product-moment correlation at time point 2 was 0.79 (*n* = 700; *p* < 0.001). Pearson's product-moment correlation at time point 3 was 0.74 (*n* = 709; *p* < 0.001). The strength of the relationship remained when data was collapsed across each of the time points (*r* = 0.76; *n* = 2247; *p* < 0.001).

### Frequency of data quality issues

#### Technical issues by time point

Frequency of technical issues (e.g., disconnection of streaming connection while video was being streamed, or Tobii not being recognized by the laptop) present at consecutive time points irrespective of camera type were 2.9, 5.5, and 3.9%, respectively. No significant differences in the frequency of technical issues were observed across the three time points (*p* > 0.1).

#### Technical issues by camera type

Analysis of technical issues associated with each camera type across the three time points revealed that of the 154 web camera-based task administrations, 7 (2.3%) had technical issues that excluded them from analysis. In contrast, 31 of the 156 eye-tracker-based task administrations (10%) had technical issues that excluded them from analysis. Chi-square analysis of the frequency of technical issues was significant (χ^2^ = 16.93; *p* < 0.001) with greater frequency of technical issues using the commercial eye tracker-based task administration.

## Discussion

This is the first study to our knowledge that has simultaneously recorded human eye movements using both a high frame rate eye tracker camera and a built-in web camera to evaluate the accuracy of the latter on a VPC decisional task in clinically normal older adults. We found that mean novelty preference scores calculated using human coding of 3 FPS data from the built-in web camera could substitute for eye movement data captured at 60 FPS using a standard eye tracker camera. The accuracy of mean novelty preference scores produced by the web camera was achieved as a result of strong inter-rater agreement of subject data at the trial level. In a subset of 25 subjects we found equivalence of mean novelty preference scores using human coding of 10 FPS data, 5 FPS, and 3 FPS data. Finally, in analysis of data quality issues associated with each camera, the built-in web camera proved more reliable across each of the three test points.

The advantages associated with the use of a built-in web camera include the ubiquity of such technology, the low cost to acquire and incorporate web cameras into task designs, and the relative ease associated with their operation and maintenance (Anderson et al., 2011). Regarding the latter, current high frame rate eye tracking cameras require extensive setup and calibration for optimal data quality, and these procedures require training and consistent implementation (Niehorster et al., 2017). Results from this study support these considerations observing higher frequency of data quality issues associated with the commercial high frame rate eye tracking camera than the built-in web camera. The technical abilities of individuals using commercial grade eye trackers also likely play a role in the frequency of technical issues. While built-in web cameras are more efficient and economical, it is important to point out that training individuals to manually score data from the web camera also has associated costs, including training time. One advantage afforded by the use of a web camera is the possibility of unobtrusive data capture of eye movements. Petridis and colleagues demonstrated a method for online acquisition of pupil size using a web camera (Petridis et al., 2013). While such applications of eye tracking may afford greater ecological validity, the clinical value of established eye tracking constructs evaluating aspects of cognition within a formal testing environment remains high.

One of the difficulties web camera eye tracking faces is the lack of clarity regarding its utility across differing task designs. Many research eye tracking task designs include measurement of numerous eye tracking features, such as gaze location, gaze fixations and re-fixations, vertical, and horizontal saccadic movements, as well as pupil measurement (e.g., diameter, dilation). The minimum frame rate required for accurate data analysis and scoring is likely quite variable and dependent upon the specific task paradigm and the specific eye tracking features of interest. While current research has demonstrated the ability to reliably capture specific features, such as gaze location and pupil diameter (Anderson et al., 2011; Petridis et al., 2013), the number of features is limited and simultaneous collection of multiple features remains a distinct advantage of commercial grade research eye trackers. In principle, the same algorithms used by research eye tracker software can be applied to extract quantitative visual feature data from web cameras. For example, identification of the pixel gradient between aspects of the eye (e.g., pupil, iris, sclera), allows for the estimation and extraction of the pupil and pupil centroid as specific shape-based features. Application of such algorithms could increase the number of features that could be simultaneously collected by a web camera.

This study provides evidence for the equivalence of scoring accuracy on a VPC decisional paradigm ranging from 3 to 60 FPS and across human and automated scoring systems. Similar studies investigating scoring equivalence across task paradigms is critical for the establishment of thresholds that can be employed when incorporating web cameras on tasks historically making use of commercial eye tracking cameras. In addition, performance metrics on eye tracking tasks can include a composite score of multiple features. For example, Lagun et al. (2011) used a combination of novelty preference, gaze fixations, saccades, and re-fixations to increase the sensitivity and specificity of a VPC task in identifying older adults with mild cognitive impairment (Cohen, 1992). The minimum frame rate required for accurate data analysis and scoring of multiple visual features is also likely dependent on the specific features of interest.

The ability to collect VPC task data remotely presents unique advantages for the assessment of memory functioning. Most measures of declarative memory require substantial instruction before administration can begin. Furthermore, verbal-based declarative memory tasks (e.g., list learning tasks) require language comprehension and non-verbal based declarative memory tasks (e.g., design learning tasks) require substantial motor output. By contrast, VPC tasks require little to no instruction, and can be provided on the screen before testing begins. VPC tasks also require minimal language comprehension or production, and minimal motor output. These factors contribute to the scalability of VPC tasks for efficient evaluation of memory function in older adults. Laske and colleagues have proposed the inclusion of eye-based screening measures within the primary care setting for earlier detection of Alzheimer's disease, and the use of a web camera could facilitate the assessment of various eye features (e.g., pupil diameter, pupil response) as well as eye-based memory function using a VPC task (Laske et al., 2015).

## Limitations and future directions

This pilot study included a small sample of clinically normal older adults. Although the sample incorporated a broad range of older ages and was ethnically diverse, the cohort was predominantly well-educated. The major limitation associated with the current method assessed is the offline nature of the system. This human-based scoring method does not allow for online tracking of eye movements, which can be incorporated into tasks through gaze-contingent designs, or immediate trial level feedback.

Additionally, the current study evaluated a VPC decisional task requiring human scorers to decide between three gaze locations (left, right, or neither). The same degree of accuracy may not be achievable on task designs that require finer discrimination of eye movements. On the other hand, Anderson and colleagues found that offline human scorers can detect and discriminate eye movements of 3° with >90% accuracy (Anderson et al., 2011). Generally speaking, the value of human scoring of web camera eye tracking data will decrease as the requirements of complexity and subtlety of eye movements increases. One important limitation of the current VPC decisional task is that it precludes analysis of more fine-grained eye tracking metrics, such as gaze fixation points, and the current method is not suitable for eye tracking paradigms requiring the collection and analysis of such metrics. As such it is important to underscore that we are not suggesting that human scoring of web camera eye tracking data can or should replace commercially available high frame rate eye trackers; instead, the results from this study suggest that specific offline gaze duration eye tracking paradigms may be more reliably and cost-effectively addressed using the approach we have detailed. Importantly, while this study examined a specific gaze duration paradigm for the evaluation of declarative memory, the same underlying paradigm has been employed across a number of developmental and neuropsychiatric conditions, including ADHD, depression, and PTSD (Armstrong et al., 2013; Isaac et al., 2014; Türkan et al., 2016). Another—somewhat obvious—limitation of this method is the requirement of human scorers to code subject eye movements. Automation of eye tracking using web camera data is a growing area of research, and open source programs exist for the automation of online, real-time eye tracking (Wang and Sung, 2001; Hansen and Pece, 2005; Li and Parkhurst, 2006; Pedersen and Spivey, 2006; Vivero et al., 2010; Krafka et al., 2016). However, the accuracy of current automated systems is below those of standard high frame rate eye tracking cameras.

This study lays the groundwork for further development in the automation of analysis and scoring of web camera-based eye tracking. We have provided evidence that for specific task designs, human scored web camera data at 3 FPS is as accurate as data obtained by a 60 FPS commercial eye tracker. The value of automated scoring of web camera data is thus dependent upon the specific task in question. In the future, we plan to compare the accuracy of an automated scoring method using web camera eye tracking data to human scored data.

## Author contributions

NB made a substantial contribution to the acquisition, analysis, and interpretation of the work, drafted the work, approved the final version to be published and agreed to be accountable for all aspects of the work. AL and DR made a substantial contribution to the conceptualization, acquisition, analysis and interpretation of the work, revised the work critically for important intellectual content, approved the final version and agreed to be accountable for all aspects of the work. EB, PC, and SZ made a substantial contribution to the analysis and interpretation of the work, revised the work critically for important intellectual content, approved the final version and agreed to be accountable for all aspects of the work.

### Conflict of interest statement

NB serves as a paid scientific consultant for Neurotrack Technologies, Inc. PC serves as a paid scientific consultant for Neurotrack Technologies, Inc. DR has served as a paid consultant for Eli Lilly, Lundbeck Pharmaceuticals and Biogen Idec. She also serves on the Scientific Advisory Board for Neurotrack Technologies, Inc. EB and SZ are co-founders of Neurotrack Technologies, Inc. Neurotrack Technologies, Inc. helped fund this study. The other author declares that the research was conducted in the absence of any commercial or financial relationships that could be construed as a potential conflict of interest.

## References

[B1] Anderson N. C. Risko E. F. Kingstone A. (2011). Exploiting human sensitivity to gaze for tracking the eyes. Behav. Res. Methods 43, 843–852. 10.3758/s13428-011-0078-8 21461633

[B2] Armstrong T. Bilsky S. A. Zhao M. Olatunji B. O. (2013). Dwelling on potential threat cues: an eye movement marker for combat-related PTSD. Depress. Anxiety 30, 497–502. 10.1002/da.22115 23620193

[B3] Bachevalier J. Brickson M. Hagger C. (1993). Limbic-dependent recognition memory in monkeys develops early in infancy. Neuroreport 4, 77–80. 10.1097/00001756-199301000-00020 8453042

[B4] Cohen J. (1992). A power primer. Psychol. Bull. 112:155. 10.1037/0033-2909.112.1.155 19565683

[B5] Crutcher M. D. Calhoun-Haney R. Manzanares C. M. Lah J. J. Levey A. I. Zola S. M. (2009). Eye tracking during a visual paired comparison task as a predictor of early dementia. Am. J. Alzheimer Dis. Demen. 24, 258–266. 10.1177/1533317509332093 19246573 PMC2701976

[B6] Fagan J. F. III. (1970). Memory in the infant. J. Exp. Child Psychol. 9, 217–226. 5452116 10.1016/0022-0965(70)90087-1

[B7] Fantz R. L. (1964). Visual experience in infants: decreased attention to familiar patterns relative to novel ones. Science 146, 668–670. 10.1126/science.146.3644.668 14191712

[B8] Gunderson V. M. Sackett G. P. (1984). Development of pattern recognition in infant pigtailed monkeys (*Macaca nemestrina*). Dev. Psychol. 20, 418–426. 10.1037/0012-1649.20.3.418 2702861

[B9] Gunderson V. M. Swartz K. B. (1985). Visual recognition in infant pigtailed macaques after a 24-hour delay. Am. J. Primatol. 8, 259–264. 10.1002/ajp.1350080309 31986814

[B10] Hallgren K. A. (2012). Computing inter-rater reliability for observational data: an overview and tutorial. Tutor. Quant. Methods Psychol. 8, 23–34. 10.20982/tqmp.08.1.p023 22833776 PMC3402032

[B11] Hansen D. W. Pece A. E. C. (2005). Eye tracking in the wild. Comput. Vis. Image Underst. 98, 155–181. 10.1016/j.cviu.2004.07.013

[B12] Isaac L. Vrijsen J. N. Rinck M. Speckens A. Becker E. S. (2014). Shorter gaze duration for happy faces in current but not remitted depression: evidence from eye movements. Psychiatry Res. 218, 79–86. 10.1016/j.psychres.2014.04.002 24751380

[B13] Knopman D. S. Roberts R. O. Geda Y. E. Pankratz V. S. Christianson T. J. Petersen R. C.. (2010). Validation of the telephone interview for cognitive status-modified in subjects with normal cognition, mild cognitive impairment, or dementia. Neuroepidemiology 34, 34–42. 10.1159/000255464 19893327 PMC2857622

[B14] Krafka K. Khosla A. Kellnhofer P. Kannan H. Bhandarkar S. Matusik W. (2016). Eye tracking for everyone, in IEEE Conference on Computer Vision and Pattern Recognition. Las Vegas, NV.

[B15] Lagun D. Manzanares C. Zola S. M. Buffalo E. A. Agichtein E. (2011). Detecting cognitive impairment by eye movement analysis using automatic classification algorithms. J. Neurosci. Methods 201, 196–203. 10.1016/j.jneumeth.2011.06.027 21801750 PMC3403832

[B16] Laske C. Sohrabi H. R. Frost S. M. Lopez-de-Ipina K. Garrard P. Buscema M.. (2015). Innovative diagnostic tools for early detection of Alzheimer's disease. Alzheimers Demen. 11, 561–578. 10.1016/j.jalz.2014.06.004 25443858

[B17] Li D. Parkhurst D. J. (eds.). (2006). Open-source software for real-time visible-spectrum eye tracking, in Proceedings of the COGAIN Conference. Turin.

[B18] Lin Y. Lin R. Lin Y. Lee G. C. (2013). Real-time eye-gaze estimation using a low-resolution webcam. Multimedia Tools Appl. 65, 543–568. 10.1007/s11042-012-1202-1

[B19] Manns J. R. Stark C. E. Squire L. R. (2000). The visual paired-comparison task as a measure of declarative memory. Proc. Natl. Acad. Sci. U.S.A. 97, 12375–12379. 10.1073/pnas.220398097 11027310 PMC17349

[B20] Niehorster D. C. Cornelissen T. H. Holmqvist K. Hooge I. T. Hessels R. S. (2017). What to expect from your remote eye-tracker when participants are unrestrained. Behav. Res. Methods 15, 1–15. 10.3758/s13428-017-0863-0 PMC5809535 28205131

[B21] Pascalis O. de Haan M. Nelson C. A. de Schonen S. (1998). Long-term recognition memory for faces assessed by visual paired comparison in 3- and 6-month-old infants. J. Exp. Psychol. Learn. Mem. Cogn. 24, 249–260. 10.1037/0278-7393.24.1.249 9438961

[B22] Pedersen B. Spivey M. (eds.). (2006). Offline Tracking of the Eyes and More with a Simple Webcam. Ithaca, NY: Cognitive Science Society.

[B23] Petridis S. Giannakopoulos T. Spyropoulos C. D. (2013). Unobtrusive low cost pupil size measurements using web cameras. NetMedicine 9–20.

[B24] Türkan B. N. Amado S. Ercan E. S. Perçinel I. (2016). Comparison of change detection performance and visual search patterns among children with/without ADHD: evidence from eye movements. Res. Dev. Disabil. 49–50, 205–215. 10.1016/j.ridd.2015.12.002 26707929

[B25] Vivero V. Barriera N. Penedo M. G. Cabrero D. Remeseiro B. (2010). Directional gaze analysis in webcam video sequences, in Lecture Notes in Compute Science: Vol. 6112, Image Analysis and Recognition, eds Campilho A. Kamel M. (Berlin: Springer), 316–324.

[B26] Wang J. G. Sung E. (2001). Gaze determination via images of irises. Image Vis. Comput. 19, 891–911. 10.1016/S0262-8856(01)00051-8

[B27] Zola S. M. Manzanares C. M. Clopton P. Lah J. J. Levey A. I. (2013). A behavioral task predicts conversion to mild cognitive impairment and Alzheimer's disease. Am. J. Alzheimer Dis. Dement. 28, 179–184. 10.1177/1533317512470484 23271330 PMC3670591

[B28] Zola S. M. Squire L. R. Teng E. Stefanacci L. Buffalo E. A. Clark R. E. (2000). Impaired recognition memory in monkeys after damage limited to the hippocampal region. J. Neurosci. 20, 451–463. 10627621 10.1523/JNEUROSCI.20-01-00451.2000 PMC6774137

